# Progress in Gastroparesis Management: From Pharmacotherapy to Interventional Treatments

**DOI:** 10.1002/jgh3.70377

**Published:** 2026-03-08

**Authors:** Ganesh Kumar, Yash Shah, Ume Rooman, Ravi Patel, Shireen Asifa, Qais M. Salah, Dushyant Singh Dahiya, Arkadeep Dhali, Camelia Arsene, Meer Ali

**Affiliations:** ^1^ Department of Medicine Chandka Medical College Larkana Pakistan; ^2^ Division of Gastroenterology and Hepatology University of Arkansas for Medical Sciences Little Rock Arkansas USA; ^3^ Department of Medicine People University of Medical and Health Sciences for Women Shaheed Benazirabad Pakistan; ^4^ Department of Internal Medicine Trinity Health Oakland/Wayne State University Pontiac Michigan USA; ^5^ Department of Medicine Dow University of Health Sciences Karachi Pakistan; ^6^ Department of Internal Medicine University of Arkansas for Medical Sciences Little Rock Arkansas USA; ^7^ Division of Gastroenterology, Hepatology & Motility The University of Kansas School of Medicine Kansas City Missouri USA; ^8^ Academic Unit of Gastroenterology Sheffield Teaching Hospitals NHS Foundation Trust Sheffield UK; ^9^ Faculty of Life Science & Medicine King's College London London UK

**Keywords:** gastric peroral endoscopic myotomy, gastroparesis, ghrelin agonists, metoclopramide, transcutaneous vagus nerve stimulation

## Abstract

Gastroparesis is a sensorimotor condition characterized by delayed gastric emptying without any obvious mechanical obstruction. Common symptoms include early satiety, nausea, vomiting, belching, and bloating. The most frequent causes of gastroparesis are diabetes, idiopathic factors, and post‐surgical complications. Currently, the only FDA‐approved medication for treating gastroparesis is metoclopramide; however, due to its potential side effects, particularly extrapyramidal symptoms, there is increasing interest in safer, more tolerable alternatives, such as prokinetics, antiemetics, and fundic relaxants. Recent advancements in pharmacological agents have demonstrated variable efficacy in improving gastric emptying and gastroparesis‐related symptoms, although symptom improvement does not consistently correlate with changes in gastric emptying metrics. In addition to pharmacological treatments, non‐pharmacological approaches like gastric peroral endoscopic myotomy (G‐POEM) and neuromodulation techniques have demonstrated significant improvements in symptoms and gastric emptying. The current understanding of gastroparesis care is still limited, and the best practices in treatment remain uncertain. Future research should prioritize multicenter trials that involve large patient populations to explore emerging therapies and innovative techniques. In this review, we will discuss the existing standards of care, advancements in novel pharmacological, interventional, and neuromodulatory treatments for gastroparesis, as well as their clinical integration, limitations, and prospects.

## 1 Introduction

Gastroparesis is characterized by delayed and slowed gastric emptying, without any mechanical obstruction causing the delay. The symptoms include nausea, vomiting, bloating, early satiety, and abdominal pain [1, 2]. Gastroparesis has a prevalence of 24.2 per 100 000 [3]. The main causes include diabetes, post‐surgical conditions, and idiopathic factors. In the recent consensus, the Gastroenterological Society of Australia (GESA) suggests that idiopathic gastroparesis is a sensorimotor disorder and affects functions beyond only gastric emptying [4]. Current treatments include dietary modifications and improving glycemic control, particularly for patients with diabetes, prokinetics, and antiemetics. Various prokinetics are available, including metoclopramide, domperidone, and macrolides, while antiemetics alleviate symptoms when initial treatments don't lead to improvement. For refractory symptoms, invasive interventions may be necessary, including decompression and feeding tubes, such as gastrostomy tubes and jejunal feeding tubes, and more aggressive treatments like pyloroplasty and endoscopic myotomy [5].

Current treatment options have limited evidence and limitations, including metoclopramide demonstrating modest efficacy and extrapyramidal effects with the extended use. Other limitations include a lack of disease‐modifying therapies and a focus on symptom control and optimization [6].

The need for new interventions for gastroparesis stems from the high prevalence of refractory symptoms and the suboptimal effectiveness of existing treatments. Newer prokinetic medications, including prucalopride, velusetrag, etc. and antiemetics like aprepitant show promise in management [7]. However, long‐term data to support their effectiveness is still needed. Additionally, emerging pyloric‐directed interventions, including gastric peroral endoscopic myotomy (G‐POEM), are showing promise for patients with refractory symptoms [7]. Neuromodulation techniques, including transcutaneous vagus nerve stimulation and electroacupuncture, have shown effectiveness and are being integrated into clinical practice [8, 9].

This review emphasizes the need for new pharmacological and interventional therapies for gastroparesis, particularly in patients with intractable symptoms. We highlight various emerging therapies and their potential, while also discussing the limitations of current treatments.

## 2 Gastroparesis: Etiology, Differentials, and Guidelines

The most common causes of gastroparesis include diabetes, post‐surgical injury, and idiopathic origins. Other potential causes include certain medications, collagen vascular disorders, thyroid issues, renal insufficiency, liver disease, and intestinal pseudo‐obstruction [10]. Gastroparesis may be associated with neurological or systemic conditions including Parkinson's disease, paraneoplastic syndromes, and scleroderma.

Recent advances in idiopathic gastroparesis have shifted its classification to a sensorimotor disorder, as outlined in GESA's new consensus. Traditional guidelines focusing on delayed gastric emptying have proven inadequate, revealing a complex interplay of factors, including gastric accommodation abnormalities, contractility issues, pyloric dysfunction, downstream dysmotility, and visceral hypersensitivity [4].

Diabetes is recognized as the primary cause of gastroparesis [5], but recent studies have indicated a potential link between obesity and gastroparesis, which may be attributed to obesity‐related inflammation and oxidative stress. Regardless, delayed gastric emptying occurs due to dysfunction in the gastric smooth muscle, interstitial cells of Cajal, or the enteric and autonomic nervous systems [11].

In patients with nausea and vomiting, consider other diagnoses such as gastroesophageal reflux disease, functional dyspepsia, gastric outlet obstruction, cannabinoid hyperemesis syndrome, rumination syndrome, cyclic vomiting syndrome, and psychological or eating disorders (Table 1) [12]. Functional dyspepsia is characterized by early satiety and often does not respond to treatment, requiring functional testing to differentiate it from gastroparesis [12, 13].

**TABLE 1:** Differential diagnosis of gastroparesis.

Condition	Similarities with gastroparesis	Differences
Functional dyspepsia and/or GERD	Postprandial fullness and early satiety, unresponsive to medical treatment	Functional testing is needed to differentiate between the two conditions
Gastric outlet obstruction	Postprandial fullness	Mechanical obstruction in the stomach or duodenum
Cyclic vomiting syndrome	Vomiting and nausea that isn't self‐induced	No other cause is found
Cannabinoid hyperemesis syndrome	Recurrent episodes of vomiting	Have normal gastric emptying between episodes, symptoms improve when cannabis use is stopped
Rumination syndrome	Undigested food is effortlessly brought back up after eating	Not preceded by nausea or vomiting
Psychiatric conditions such as anxiety disorders, anorexia nervosa, and bulimia	Nausea or vomiting, weight loss	Associated psychiatric problems

Gastric outlet obstruction mimics other conditions but involves mechanical blockage (Table 1) [12]. Cyclic vomiting syndrome presents with recurrent, non‐inducible vomiting, weekly for a minimum of three months, without any identifiable causes (Table 1) [13]. Rumination syndrome is a behavioral condition defined by the effortless regurgitation of undigested food after meals, without any preceding nausea (Table 1) [12]. Cannabinoid hyperemesis syndrome occurs in marijuana users, experiencing cyclic vomiting but having normal gastric emptying between episodes. Symptoms improve once cannabis use is discontinued (Table 1) [12, 13]. Additionally, psychiatric conditions including anxiety disorders, anorexia nervosa, and bulimia may present with nausea and vomiting (Table 1) [12, 13].

The gold standard for diagnosing gastroparesis is gastric emptying scintigraphy with a radio‐labeled solid meal, offering a non‐invasive measurement [10]. An FDA‐approved alternative is the ^13^C breath test using spirulina algae, which is simple, cost‐effective, and does not require on‐site equipment; breath samples can be analyzed later [5]. However, the ^13^C test has limitations: it cannot identify the specific affected gastric region and may yield false positives in patients with lung or liver diseases. It is also important to rule out gastric outlet obstruction before the test [14].

## 3 History and Evolution of Gastroparesis Management

Since the 20th century, gastroparesis management has relied on dietary changes, emphasizing small, low‐fat, and low‐fiber meals. For those intolerant to solid foods, liquid or finely ground diets can improve gastric emptying and reduce symptoms. Although the relief provided was modest [15, 16, 17, 18]. A trial with diabetic gastroparesis patients found that a reduced‐particle diet lowered symptom severity compared to a standard diet [18].

By the early 2000s, pharmacologic therapy became essential for individuals with persistent symptoms despite dietary changes [19]. Prokinetics, which improve antral contractions and enhance food emptying, emerged as the standard treatment, recommended 10 to 15 min before meals and at night for nocturnal symptoms [15]. Liquid formulations were later introduced for precise dosing and improved absorption, leading to enhanced symptom relief and tolerability.

Metoclopramide was the first effective prokinetic medication that improved both solid and liquid gastric emptying, reducing refractory symptoms by nearly 30% in early trials [15]. Its use is limited due to extrapyramidal side effects, leading to a 2009 FDA boxed warning advising treatment not to exceed 12 weeks unless benefits outweigh risks [20]. Studies in Scandinavia and the UK indicated a risk of irreversible tardive dyskinesia of less than 0.1% per 1000 patient‐years, prompting cautious prescribing practices such as informed consent, “drug holidays,” and quarterly neurologic monitoring [20, 21]. While metoclopramide remains a key therapy, it is now used more cautiously. An intranasal spray was introduced in the 2010s for patients who cannot tolerate pills, but a trial showed symptom relief in women with diabetic gastroparesis, emphasizing the need for further studies [22].

Domperidone, introduced in the 1970s, is a peripheral D_2_‐receptor antagonist that effectively treats diabetic gastroparesis without central nervous system side effects [23, 24]. Although its efficacy is similar to metoclopramide, concerns about QT prolongation prevented FDA approval in the US, so it's available only through Investigational New Drug protocols for those intolerant to metoclopramide [15]. In Europe, Canada, and Asia, domperidone is widely used, aided by guidelines that manage cardiac risks. A network meta‐analysis has shown that domperidone and clebopride are among the top prokinetics for symptom relief [25].

In the 1980s, cisapride, a selective 5‐HT_4_ agonist, was noted for enhancing motility and normalizing gastric emptying, but it was withdrawn from the US market due to serious drug interactions, including fatal arrhythmias. Newer 5‐HT_4_ agonists like prucalopride have emerged, showing improved safety and effectiveness in speeding up gastric emptying in healthy individuals and alleviating symptoms in idiopathic gastroparesis. However, prucalopride was not more effective than placebo for diabetic or connective tissue‐related gastroparesis, underscoring the need for safe prokinetic options.

In the 1990s, macrolide motilin agonists like erythromycin offered a new approach to treating gastroparesis, but their use was limited by tachyphylaxis and side effects. Azithromycin, another macrolide with fewer gastrointestinal issues and a longer half‐life, also posed cardiovascular risks.

In the 2000s, cannabinoids were explored but none received US approval, except for cannabidiol (Epidiolex), which shows promise in treating gastroparesis [26]. In the 2010s, neurokinin‐1 antagonists developed for chemotherapy‐induced nausea had limited benefits [27]. Low‐dose nortriptyline showed potential in open‐label studies for nausea and abdominal pain but did not outperform placebo. Mirtazapine has emerged as a promising alternative, showing preliminary antiemetic benefits in gastroparesis [28].

Procedural interventions were introduced for patients unresponsive to prokinetic and antiemetic treatments, including percutaneous endoscopic gastrostomy (PEG) and jejunostomy (PEJ) for gastric decompression and nutrition. As pharmacologic and nutritional therapies proved insufficient, targeted pyloric interventions were developed to address gastric outflow resistance [29, 30]. In the 2000s, laparoscopic pyloroplasty became the first durable surgical option, providing sustained symptom relief and accelerated gastric emptying [31]. Endoscopic approaches like transpyloric stents showed about a 75% clinical response in refractory cases, particularly for nausea and vomiting [32]. Gastric peroral endoscopic myotomy (G‐POEM) has recently gained attention, showing high technical success and significant GCSI score reductions, with a sham‐controlled trial revealing 71% treatment success versus 22% for controls [33, 34, 35, 36]. These advances have overtaken intrapyloric botulinum toxin, which was abandoned after two trials failed to show superiority over placebo [36, 37], and relegated gastric electrical stimulation to a role for humanitarian use in the most refractory cases [38].

Table 2 contrasts key recommendations from American [15, 16] and European [39] gastroparesis guidelines, illustrating how therapeutic advancements have been integrated into each framework.

**TABLE 2:** American [15, 16] and European [39] gastroparesis guidelines.

Domain	ACG (USA) guideline (2022)	UEG/ESNM (Europe) consensus (2021)
Definition	Gastroparesis = symptoms with delayed emptying of solids ≥ 10% retention at 4 h; no mechanical obstruction.	Epigastric symptoms (especially nausea and vomiting) with delayed gastric emptying; no obstruction.
Diagnosis	Gold standard: gastric scintigraphy (3–4 h); breath test and wireless capsule as alternatives.	Mandatory: Endoscopy + validated GE test (scintigraphy or breath test); wireless capsule not supported.
Dietary Therapy	Small‐particle, low‐fat diets and frequent meals recommended (conditional).	Agreed that dietary modification improves symptoms and emptying.
Prokinetics	Metoclopramide (12‐wk limit), domperidone (IND only), prucalopride emerging (conditional).	Only dopamine‐2 antagonists and 5‐HT_4_ agonists endorsed.
Antiemetics	Symptom control only	No consensus beyond D_2_ or 5‐HT_4_ agents.
Neuromodulators	Not recommended, mirtazapine used off‐label	No support for TCAs, SSRIs, or other neuromodulators.
Rescue motilides	Erythromycin and azithromycin considered for short‐term use; concern for tachyphylaxis.	No consensus: concerns over side effects remain.
Nutritional support	PEG for venting; PEJ for nutritional support; TPN only for gastric–small bowel failure.	Agreed: enteral/parenteral nutrition for severe weight loss or vomiting.
Pyloric‐targeted therapies	Botulinum toxin not recommended; pyloromyotomy (lap or G‐POEM) reserved for refractory cases.	Botulinum toxin not supported; G‐POEM and pyloroplasty considered after failed medical therapy.
Gastric electrical stimulation	HUD device; may reduce vomiting frequency (conditional).	No consensus; mixed data in trials.
Follow‐up and prognosis	Symptom monitoring with GCSI.	Prognosis varies by etiology; no agreement on life‐expectancy impact.

## 4 Recent Advances in Pharmacological Therapy

### 4.1 Prokinetics

The only FDA‐approved drug for gastroparesis is metoclopramide, which is limited due to its side effects. There is growing interest in safer alternatives, particularly prokinetics that enhance gastric motility. Ghrelin agonists like relamorelin have shown promise in stimulating gastric motility, but clinical responses are mostly symptom‐based and don't always align with objective measures of gastric emptying (Table 3). Newer 5‐HT_4_ receptor agonists, such as prucalopride, velusetrag, and felcisetrag, show better safety and efficacy for treating gastroparesis [40]. It is increasingly recognized that improvements in gastric emptying do not reliably predict symptom relief in gastroparesis, underscoring the multifactorial pathophysiology of the disease and the importance of symptom‐directed therapeutic strategies [4, 15].

**TABLE 3:** Summary of prokinetics for gastroparesis.

Drug	Dosing	Formulation	Unique quality
Relamorelin	100 μg	Subcutaneous	No cardiovascular or extrapyramidal adverse effects
Prucalopride	2–4 mg	Oral	No cardiac adverse effects
Velusetrag	5, 15 and 30 mg	Oral	Tolerance to side effects may develop
Felcisetrag	0.1, 0.3 and 1 mg	Intravenous	A single 0.5 mg IV dose was found to be as effective as multiple 10 mg doses of metoclopramide

*Note:* With the exception of metoclopramide, which is FDA‐approved for gastroparesis, pharmacological agents listed are investigational or used off‐label. Endoscopic and neuromodulatory therapies are considered adjunctive or investigational and are not uniformly endorsed by current clinical guidelines.

### 4.2 Relamorelin

Relamorelin is a synthetic ghrelin agonist that is 15 to 30 times more potent than natural ghrelin (Table 3) [41]. It stimulates contractions in the gastric body and antrum, aiding in gastric emptying [13]. In patients with diabetic gastroparesis, relamorelin significantly speeds up gastric emptying of solids and reduces symptoms like nausea, vomiting, bloating, and abdominal pain, although these improvements do not always correlate with the speed of gastric emptying [4, 15, 40]. In healthy individuals, it increases antral contractions without affecting gastric accommodation or postprandial satiety [42]. Additionally, relamorelin has no cardiovascular or extrapyramidal side effects, making it suitable for those with diabetic gastroparesis and constipation [41].

In studies, a subcutaneous dose of 100 μg significantly accelerated gastric emptying in diabetic patients (Table 3) [43, 44]. Phase IIa and IIb trials showed promising results for relamorelin in patients with diabetic gastroparesis. The Phase IIa trial with a 10 μg subcutaneous dose twice daily demonstrated a significant reduction in gastric emptying half‐time and improvement in symptoms [44]. The Phase IIb trial, using a 100 C dose once daily, reported a 13‐min reduction in T1/2 and a 75% decrease in vomiting frequency [45]. Barring postprandial hyperglycemia in 14.5% of patients, the drug was well tolerated [45].

A 2023 meta‐analysis with 1033 participants found that relamorelin improved T1/2 by a mean of 11.40 min compared to placebo, with an 8.43‐min improvement in diabetic gastroparesis [46]. Adverse effects included headaches, dizziness, and gastrointestinal symptoms, with mild diarrhea reported due to ghrelin receptor activity [46].

### 4.3 5‐HT_4_ Receptor Agonists (Prucalopride and Velusetrag, and Felcisetrag)

New generation 5‐HT_4_ agonists, including prucalopride, velusetrag, and felcisetrag, demonstrate high receptor selectivity and show minimal affinity for other serotonergic or non‐serotonergic receptors. These agonists impact potassium current associated with arrhythmias only at very high concentrations, about 300 times higher than that of cisapride, providing a significant cardiac safety [40].

### 4.4 Prucalopride

Prucalopride is approved for chronic constipation due to its enterokinetic properties. Recent trials have demonstrated that it exhibits gastrokinetic properties and improves symptoms in idiopathic gastroparesis [5]. This suggests that prucalopride could be particularly beneficial for both gastroparesis and colonic inertia [14].

Two 4‐week trials evaluated the effects of prucalopride at daily doses of 1–4 mg, revealing improvements in gastric emptying and reduction in symptoms as measured by the GCSI (Table 3). Headache and mild GI adverse effects were reported (Table 3) [47].

Carbone et al. and Andrews et al. studied prucalopride over 4 weeks with daily doses of 2 mg and 4 mg, respectively [48, 49]. Carbone et al. reported significant symptom improvement in the idiopathic gastroparesis subgroup with the 2 mg dose. Conversely, Andrews et al. found that the 4 mg dose improved gastric emptying and bowel movement frequency but did not relieve symptoms in diabetic gastroparesis [48]. This lack of symptom improvement, despite enhanced gastric emptying, aligns with existing evidence and reinforces the concept that delayed gastric emptying is an imperfect surrogate for symptom severity in gastroparesis. Janssen et al. also noted no consistent relationship between gastric emptying time reduction and symptom relief in patients on prokinetic therapy, suggesting that rapid gastric emptying might reduce fundic accommodation and provoke symptoms, while gastrointestinal side effects could mask persistent symptoms [48].

In Carbone et al. and Andrews et al., minor side effects including headaches, dizziness, and abdominal cramps were noted in patients receiving prucalopride, and one patient developed small bowel volvulus [48, 49].

Prucalopride is safer than metoclopramide. The FDA identified 1085 reports on metoclopramide, with the most common adverse effects including tardive dyskinesia, dystonia, QTc prolongation, Torsade de Pointes, and pheochromocytoma crisis. In comparison, among 865 reports on prucalopride, the most frequently reported side effects were headaches, diarrhea, and abdominal pain, alongside few rare reports of dystonia [50].

### 4.5 Velusetrag

Velusetrag is a 5‐HT_4_ receptor agonist used to treat bloating and early satiety, though clinical results have been mixed. In patients with chronic idiopathic constipation, doses of 15, 30, and 50 mg daily over 4 weeks expedited gastrointestinal emptying within 4–9 days (Table 3). While it remains investigational for gastroparesis, studies show improvements in gastric emptying but inconsistent symptom relief [11].

Research by Kuo et al. and Abell et al. evaluated velusetrag at 5, 10, and 30 mg doses over 1–3 weeks. Kuo et al. found all doses improved gastric emptying time (GET), with the 30 mg significantly reducing GET by ≥ 20% in many patients, showing comparable results for both diabetic and idiopathic gastroparesis [51]. Abell et al. reported that about 70% of patients receiving 30 mg normalized GET at 4 h, while symptom improvement was noted only in idiopathic patients on the 5 mg dose [52]. Ahn et al. confirmed these findings and indicated that 30 mg improved GET in both subgroups [11].

No serious adverse effects have been linked to velusetrag. Kuo et al. noted that higher doses yielded fewer GI side effects, indicating potential desensitization [51]. However, Abell et al. observed a dose‐dependent increase in GI side effects, likely from off‐target stimulation of colonic motility [52]. A systematic review mentioned possible QTc prolongation, but no extrapyramidal side effects were observed (Table 3) [48].

### 4.6 Felcisetrag

Felcisetrag is a 5‐HT_4_ receptor agonist being studied for its impact on gut motility, with cardiac safety still under evaluation. A study by Chedid et al. involved gastroparesis patients receiving daily intravenous doses of 0.1, 0.3, and 1 mg for 3 days (Table 3). Results indicated a dose‐dependent improvement in gastric emptying time compared to placebo, with no significant abnormalities in lab results, vital signs, or ECGs [53].

In another interesting trial by Chapman et al., a single intravenous dose of 0.5 mg of felcisetrag was found to be as effective as multiple 10 mg doses of metoclopramide in accelerating gastric emptying, without increasing the risk of adverse events [54].

Regarding cardiac safety, an in vivo study by Beattie et al. indicated that both velusetrag and felcisetrag did not have significant effects on coronary tone or cardiac rhythm, as assessed via hERG potassium channels, and there were no observable impacts on other target sites [55]. Although a systematic review and meta‐analysis (SRMA) from 2024 reported QTc prolongation associated with these agents [48], it noted that none of the prolonged QTc intervals were clinically significant or exceeded 500 millisecon [51, 53].

### 4.7 Other Drugs

#### 4.7.1 Trazpiroben

Trazpiroben (TAK‐906) is an investigational peripheral acting, selective dopamine D_2_/D_3_ receptor antagonist, currently under evaluation for symptom management in gastroparesis (Table 4). Unlike metoclopramide and domperidone, it has a limited penetration into the brain and a low affinity for hERG potassium channels, which equates with a reduced risk of CNS and CVS side effects [56].

**TABLE 4:** Summary of emerging pharmacological therapies.

Drug	MOA	Phase of development	Efficacy	Adverse effects
Trazpiroben	Selective dopamine D2/D3 receptor antagonist	Phase 1 and 2 trials	No clinically meaningful difference in efficacy between trazpiroben and placebo, but has shown benefits in volume‐to‐fullness	Headache, dizziness, presyncope, fatigue
Buspirone	5‐HT_1_A receptor agonist	Phase 1	It may help reduce symptoms like early satiety and bloating	Vomiting, diarrhea, drowsiness, headache, vertigo
Tradipitant	Neurokinin‐1 (NK1) receptor antagonist	Phase 1, 2, and 3 trials	Shows a reduction in nausea severity and more nausea‐free days. There may be a potential dose‐related effect on gastric motor function	Diarrhea, urinary tract infections, headache
Cannabidiol	Inverse agonist at CB2 (Cannabinoid type 2) receptors	Phase 1 and 2 trials	Subjects had fewer vomiting episodes and improved ability to finish meals	Diarrhea, headache, nausea, fatigue

*Note:* With the exception of metoclopramide, which is FDA‐approved for gastroparesis, pharmacological agents listed are investigational or used off‐label. Endoscopic and neuromodulatory therapies are considered adjunctive or investigational and are not uniformly endorsed by current clinical guidelines.

Whiting et al. reported that trazpiroben showed rapid absorption with a median *T*_*max*_ of 1.1 h and a half‐life of 4 to 11 h following doses of 5–300 mg or multiple doses of 50–100 mg. The drug was well‐tolerated, with only mild adverse effects like headache, dizziness, presyncope, and fatigue. No significant changes were observed in lab values, vital signs, or cardiac assessments, and QTc changes remained minimal, indicating a favorable cardiac safety profile across all doses [56].

A trial by Kuo et al. found no improvement in gastric emptying time (GET) with trazpiroben or metoclopramide. However, trazpiroben at 5 and 25 mg showed improvements in volume‐to‐fullness, with a nonsignificant numerical benefit at 25 mg [57]. Likewise, Tack et al. reported no significant differences between the placebo and trazpiroben groups on the Gastroparesis Cardinal Symptom Index, though trazpiroben was well tolerated [58]. A study in a Japanese population confirmed similar pharmacokinetic and safety results, indicating consistency across ethnic groups [41].

#### 4.7.2 Buspirone

Buspirone, a serotonergic 5‐HT_1_A receptor agonist, has been shown to promote relaxation of the gastric fundus. It may also help relieve gastroparesis symptoms such as bloating and early satiety (Table 4) [41]. Buspirone may be especially useful in patients with gastroparesis with overlapping anxiety or functional dyspepsia, as it has both gastric and anxiolytic effects [14]. Tack et al. reported that buspirone (10 mg, TDS) for 4 weeks daily showed a considerable reduction in postprandial fullness, early satiety, and upper abdominal pain [59].

A 4‐week trial of buspirone (10 mg, TDS) for moderate to severe postprandial fullness showed no significant improvement in primary symptom scores compared to placebo. However, patients with severe baseline bloating experienced noticeable relief. Adverse effects included nausea, vomiting, diarrhea, and drowsiness. Further research is needed on buspirone's efficacy for gastroparesis, particularly for those with predominant bloating or impaired fundic accommodation, with longer assessment periods recommended [60].

#### 4.7.3 Tradipitant

Tradipitant is a Neurokinin‐1 (NK1) receptor antagonist that selectively blocks substance P (Table 4). It has minimal affinity for other receptors like serotonin or dopamine. Currently, tradipitant is used with ondansetron to manage chemotherapy‐induced nausea. Recent evidence suggests it may also alleviate symptoms of gastroparesis and improve fundic accommodation, expanding its potential uses beyond chemotherapy‐related nausea [14].

In a trial with predominantly female patients suffering from moderate to severe nausea due to idiopathic (60%) or diabetic gastroparesis (40%), tradipitant significantly reduced nausea severity and increased nausea‐free days. It also improved secondary endpoints like Patient Assessment of Gastrointestinal Disorders Symptom Severity Index (PAGI‐SYM) [5].

A study by Khanna et al. with healthy volunteers tested tradipitant (85 mg, oral, BD) over 9 days and found no significant effects on gastric emptying, volume, or postprandial symptoms compared to placebo. However, higher blood levels of tradipitant correlated with improved gastric accommodation and faster gastric emptying at 4 h, indicating a potential dose‐related effect on gastric motor function [61].

Carlin et al. found that tradipitant significantly reduced nausea and improved symptoms in diabetic and idiopathic gastroparesis over a 4‐week period, particularly in those with baseline nausea and vomiting [62]. A larger phase 3 trial in 2023 with 152 patients further confirmed these results, showing considerable reductions in nausea severity and more nausea‐free days [63]. About half of the tradipitant group had a ≥ 1‐point improvement on the GCSI. Both trials reported rare adverse effects, including diarrhea, UTIs, dizziness, and headaches, occurring at similar rates in the placebo and tradipitant groups [62, 63].

#### 4.7.4 Cannabidiol

The role of medical marijuana in gastroparesis is currently under investigation. In clinical settings, cannabinoids are already used to manage chronic pain, sleep disorders, spasticity, chemotherapy‐induced nausea and vomiting, as well as HIV‐associated weight loss. However, emerging data suggests a potential use of cannabinoids in gastrointestinal disorders, including gastroparesis (Table 4) [64]. A study reported that patients with gastroparesis who were using cannabis had shorter hospital stays and lower inpatient mortality [64]. Although another questionnaire‐based study found no significant difference in gastric emptying between users and non‐users but reported improved symptom control in patients using cannabinoids [64].

Cannabidiol (CBD) acts as an inverse agonist at CB2 receptors and has shown potential in modulating intestinal inflammation (Table 4) [65]. Zhang et al. investigated the FDA‐approved formulation of cannabidiol (20 mg/kg/day) in 44 patients with gastroparesis, with documented delayed gastric emptying [65]. While cannabidiol was found to further slow down gastric emptying, patients also demonstrated significant clinical improvements, including lower GCSI‐Daily Diary scores, fewer episodes of vomiting, and improved ability to finish meals. Additionally, cannabidiol also significantly increased calorie intake and maximum tolerated intake during a nutrient drink test. Side effects like nausea, diarrhea, fatigue, and headache were reported but were generally mild [65]. However, the therapeutic potential of cannabidiol depends significantly on appropriate dosing, which should be properly determined in large randomized controlled trials [14].

## 5 Endoscopic Intervention—Gastric Peroral Endoscopic Myotomy (G‐POEM)

Gastric peroral endoscopic myotomy (G‐POEM) is an endoscopic procedure utilized for patients with refractory gastroparesis who have failed medical therapy, including prokinetic medications and other less invasive therapies available. The procedure involves creating a submucosal tunnel in the gastric antrum and performing a myotomy of the pyloric sphincter, which enhances gastric emptying.

The technique of performing G‐POEM involves creating a submucosal bleb about 4–5 cm proximal to the pylorus, typically along the greater curvature. A longitudinal or transverse 1.5–2‐cm mucosal incision is then made, and the endoscope is introduced into the submucosal space, thus creating a tunnel toward the pyloric ring where, once reached, a myotomy is performed [26].

Clinical outcomes are favorable for G‐POEM with a randomized controlled trial showing a clinical success (≥ 50% reduction in GCSI) in 71% of patients at 6 months follow‐up, compared to 22% with sham (*p* = 0.005), with the highest response evident in diabetic gastroparesis. Gastric emptying studies and symptom scores both improved post‐procedures [66]. A meta‐analysis of a total of 20 studies has shown clinical success, with mean preprocedural GCSI scores of 3.38 ± 0.37, which have improved remarkably postprocedural [weighted mean difference −1.56 (95% CI: −1.89 to −1.24); *p* < 0.001] [34].

Despite promising results of G‐POEM, multiple limitations must be considered. Patients' heterogeneous responses tend to be more effective in diabetic and idiopathic gastroparesis compared to postsurgical gastroparesis. Long‐term outcome data are still missing, and long‐term durability of symptom relief postprocedural is still being evaluated [34].

## 6 Neuromodulation and Alternative Therapies

### 6.1 Electroacupuncture/Acupuncture

Electroacupuncture (EA) and traditional acupuncture are promising non‐drug interventions for managing gastroparesis, especially in patients who do not respond well to conventional therapies (Table 5) [36, 67, 68]. These methods involve inserting needles at specific acupoints, particularly ST36 (Zusanli), along with PC6 (Neiguan), RN12 (Zhongwan), and BL21 (Weishu). EA adds a low‐intensity electrical current to enhance neuromodulation. This approach has been effective for various types of gastroparesis, including idiopathic, diabetic, and postsurgical forms, particularly when conventional treatments are inadequate or when patients seek alternative therapies [36, 67].

**TABLE 5:** Summary of the Neuromodulatory and Interventional therapies.

Intervention	Mechanism	Outcomes/benefits	Adverse outcomes or risks	Cost
Electroacupuncture (EA)	Peripheral nerve stimulation at acupoints (e.g., ST36, PC6) modulates enteric and central autonomic pathways; enhances vagal tone and gastrointestinal motility	– Improved gastric emptying – Reduced nausea, vomiting, bloating, and abdominal pain – Enhances gastric slow‐wave activity, and possibly GI hormone regulation	– Mild skin irritation – Needle discomfort – Rare bruising – Vasovagal reaction	Generally low; varies by region and provider. Cost‐effective adjunctive therapy
tVNS	Non‐invasive electrical stimulation of auricular or cervical vagus branches; enhances parasympathetic output, modulates gut‐brain axis, may reduce inflammation	– Reduces GCSI scores – Improves nausea, vomiting, early satiety, gastric emptying in responders – Promising alternative for refractory cases	– Mild skin discomfort at electrode site – Rare transient headache or dizziness – No systemic adverse effects reported	Moderate, dependent on device used (gammaCore, tVNS). Some may require prescription or clinical supervision

RCTs and meta‐analyses show that EA significantly enhances gastric emptying, alleviates symptoms like nausea and bloating, and stabilizes gastric slow wave activity, particularly at ST36 (Table 5) [67, 68]. Benefits are observed in both animal models and patients, improving key symptoms of gastroparesis with mild, infrequent adverse effects. Combining EA with the prokinetic drug mosapride may benefit patients with severe gastroparesis [36].

EA/Acupuncture is becoming more accessible worldwide due to expanded practitioner training and growing acceptance in Western medicine. It is considered safe, cost‐effective, and low risk, making it an appealing option for managing symptoms comprehensively.

### 6.2 Transcutaneous Vagus Nerve Stimulation

Transcutaneous vagus nerve stimulation (tVNS) is a non‐invasive neuromodulation technique (Table 5). tVNS is indicated for patients with persistent idiopathic or diabetic gastroparesis despite standard pharmacotherapy and as alternatives to invasive procedures (Table 5) [69]. tVNS delivers mild electrical pulses to the auricular branch or the cervical branch of the vagus nerve by surface electrodes. This procedure allows self‐administration, using hand‐held devices, and is well tolerated with a few to several minutes sessions per day over sustained periods (Table 5).

tVNS may modulate neural circuits related to gastrointestinal motility and sensation. In a pilot study of idiopathic gastroparesis patients, self‐administered cervical stimulation with gammaCore for 4 weeks resulted in significant improvements in GCSI scores, with 40% meeting the responder threshold. Some patients also showed faster gastric emptying (T1/2 reduced from 155 to 129 min; *p*≈0.05) [69]. tVNS has been shown to modulate gastric myoelectric activity and increase gastrin levels in perioperative settings, indicating that efferent vagal activation affects gut function [70].

tVNS is a non‐invasive procedure that avoids risks associated with surgically implanted devices, like infections. It selectively activates vagal fibers for gastrointestinal regulation while preserving other neural pathways and allows for customizable parameters such as frequency, amplitude, and duration to suit individual patient needs [69, 71]. Adverse events include local skin irritation, tingling, or transient neck discomfort, with no serious effects (Table 5) [71]. Several non‐invasive tVNS devices have been evaluated in gastroparesis and related conditions:

1. gammaCore (electroCore, USA) is a hand‐held device that delivers electrical stimulation to the vagus nerve in the neck. It is FDA‐cleared for migraine and cluster headache and has been studied off‐label for gastroparesis, showing response rates of 35%–45% in small cohorts over 3–6 weeks. Side effects were minor, like neck discomfort or skin irritation, with no serious adverse events reported [69, 72].
2. Auricular tVNS devices are clip‐on stimulators that target the auricular branch of the vagus nerve in the ear's cymba concha or tragus. Their proximity to vagal afferents enables effective neuromodulation. In clinical trials with healthy volunteers, these devices showed real‐time modulation of gastric motility and reduced inflammatory markers [73].
3. Recent advancements in tVNS technology are aimed at enhancing usage, personalization, and patient comfort. New designs feature wearable neckbands and Bluetooth‐enabled auricular patches that allow users to adjust stimulation via smartphone apps. While large‐scale clinical evidence in gastroparesis is still pending, these wireless, app‐driven devices signify a trend toward personalized, home‐based neuromodulation [74].

## 7 Clinical Integration

The 2022 ACG Clinical Guideline highlights best practices for diagnosing and managing gastroparesis, while also noting several emerging treatments, primarily investigational or used off‐label, that may offer symptom relief for refractory cases. Although these agents, including ghrelin agonists and selective 5‐HT_4_ receptor agonists, are not formally recommended, they present evolving options that target specific symptoms. Their integration into standard care requires careful evaluation due to the lack of confirmatory evidence and formal guidelines; metoclopramide remains the primary approved option among them.

Trazpiroben (TAK‐906) is a peripherally selective dopamine D_2_/D_3_ antagonist that, while not included in the ACG guideline, may be suitable for those at risk of CNS or cardiac side effects. Early trials indicate modest symptom relief without significantly affecting gastric emptying, suggesting it's best used with prokinetics or antiemetics for patients mainly experiencing early satiety or bloating rather than nausea or vomiting [75].

Tradipitant, a neurokinin‐1 receptor antagonist with central antiemetic properties, was noted in the ACG document despite not meeting its primary nausea endpoint in initial studies. However, it showed significant reductions in vomiting frequency, postprandial fullness, and GCSI scores, suggesting its potential as an adjunct treatment for idiopathic gastroparesis if it receives FDA approval [15, 76].

Cannabidiol (Epidiolex), a low‐THC formulation approved for seizure disorders, shows encouraging effects on vomiting and nutrient tolerance despite a paradoxical slowing of gastric emptying. These findings hint at a central neuromodulatory action that benefits patients with intractable vomiting who cannot tolerate standard antiemetics. Its high cost and regulatory hurdles may limit widespread adoption [26].

Buspirone, a 5‐HT_1_A agonist noted briefly in the ACG guideline may alleviate early satiety by enhancing fundic accommodation. Though not formally recommended, its generic status and favorable safety profile make it an attractive off‐label option for patients with primary complaint of postprandial fullness [63].

Electroacupuncture carries a conditional recommendation for diabetic gastroparesis based on low‐quality evidence. Small studies suggest that, when paired with pharmacotherapy, transcutaneous electroacupuncture improves nausea, bloating, and gastric accommodation, offering a low‐risk adjunct.

tVNS is not formally recommended but shows potential benefits for idiopathic gastroparesis, improving gastric motility and reducing nausea and GCSI scores. As a non‐invasive therapy, it may be ideal for patients who cannot undergo invasive procedures; however, larger trials are needed to establish optimal parameters and cost‐effectiveness [69].

These agents highlight a shift toward personalized therapy for gastroparesis. Their clinical integration will rely on future evidence, regulatory approval, and considerations of symptom dominance, such as nausea or impaired motility. While combining them with first‐line drugs is often feasible, caution is needed to avoid counteracting motility. Additionally, cost, accessibility, and reimbursement will influence their practicality in various care settings.

## 8 Limitations

Advancements in treatments for gastroparesis, including 5‐HT_4_ agonists, dopamine antagonists, gastric electrical stimulation, G‐POEM, EA, and tVNS, face limitations due to insufficient evidence. Many studies have small sample sizes and lack control groups, hindering generalizability. Cohorts examining neuromodulation techniques like tVNS or electroacupuncture often involve fewer than 20 participants and lack long‐term follow‐up. While meta‐analyses suggest symptomatic benefits, they highlight substantial heterogeneity, regression to the mean, and publication bias, emphasizing the need for large‐scale, high‐quality RCTs with proper design [77].

There is a lack of multicenter RCTs comparing novel therapies, like 5‐HT_4_ agonists, with standard treatments such as G‐POEM or tVNS. No Phase IV data exist for long‐term safety, and pediatric and pregnant populations are underrepresented, with only 12 small retrospective pediatric RCTs identified and no standardized guidelines for interventions [78]. A recent review confirms that pregnant and breastfeeding women are excluded from trials on gastroparesis treatments, due to which no standard treatment exists for managing them [79].

## 9 Future Directions

Advancements in gastroparesis management need wider access to interventional therapies and multicenter trials to evaluate optimal treatments, such as recent comparisons of G‐POEM with pyloric botulinum toxin injections and jejunal feeding [80, 81]. For optimal clinical decisions, research must prioritize trials with direct comparisons of drugs to procedural approaches.

Uniform standardization of outcome measures is needed, as studies use a mix of methods like gastric emptying scintigraphy and symptom tools such as the GCSI. A uniform composite endpoint would enhance comparability and meta‐analytic accuracy [82]. The integration of digital health technologies with neuromodulation techniques is transforming personalized care. Preliminary data indicate that Bluetooth‐enabled, wearable tVNS systems enable real‐time symptom tracking and adaptable home‐based therapy.

## 10 Conclusion

Gastroparesis management is entering a pivotal era driven by advances in pharmacological approaches, minimally invasive endoscopic procedures, and cutting‐edge neuromodulation techniques. Highly targeted therapies are redefining the pharmacotherapeutic guidelines by offering symptom control with improved safety. These promising investigational and off‐label therapies may influence future guidelines pending further high‐quality RCTs and approval. Interventions like G‐POEM, EA, and tVNS show positive outcomes, extending non‐invasive options for resistant cases or those seeking alternatives. The integration of digital health with AI‐assisted diagnostics and real‐time gastric motility monitoring advances personalized care. However, gaps remain, including the lack of multicenter RCTs comparing modalities, limited long‐term safety data, and the inclusion of pediatric and pregnant populations in trials. These advances shift focus from symptom management to disease modification. As high‐quality trials clarify comparative effectiveness, durable, patient‐centered gastroparesis management becomes attainable, offering hope for improved quality of life.

## Funding

The authors have nothing to report.

## Ethics Statement

The authors have nothing to report.

## Consent

The authors have nothing to report.

## Conflicts of Interest

The authors declare no conflicts of interest.

## Data Availability

Data sharing not applicable to this article as no datasets were generated or analyzed during the current study.
